# Defining successful program configurations in VA home-based primary care: a study protocol to identify key difference-makers through investigating cross-case heterogeneity in program implementation

**DOI:** 10.1186/s12877-025-06502-7

**Published:** 2025-11-25

**Authors:** Anaïs Tuepker, Edward J. Miech, Elizabeth C. Hulen, Meike Niederhausen, Catherine S. Hwang, Bruce Kinosian, Allison O’Neill, Senta G. Wiederholt, Chandra Penn, Dayna Cooper, Samuel T. Edwards

**Affiliations:** 1 Center to Improve Veteran Involvement in Care, VA Portland Health Care System, 3710 SW U.S. Veterans Hospital Rd, R&D 66, Portland, OR 97239 USA; 2 Department of Family Medicine, Oregon Health & Science University, Portland, OR USA; 3 Indiana University School of Medicine, Indianapolis, IN USA; 4 Veterans Rural Health Resource Center, VA Office of Rural Health, Portland, OR USA; 5 OHSU-PSU School of Public Health, Oregon Health & Science University, Portland, OR USA; 6 Section of General Internal Medicine, VA Portland Health Care System, Portland, OR USA; 7 Division of General Internal Medicine and Geriatrics, Oregon Health & Science University, Portland, OR USA; 8 Geriatrics and Extended Care Data Analysis Center (GECDAC), Cpl. Michael J Crescenz VA Medical Center, Philadelphia, PA USA; 9 Center for Health Equity Research and Promotion, Cpl. Michael J Crescenz VA Medical Center, Philadelphia, PA USA; 10 Department of Medicine, University of Pennsylvania, Philadelphia, PA USA; 11 Office of Geriatrics and Extended Care, Veterans Health Administration, Washington DC, USA

**Keywords:** Primary care, Geriatrics, Program evaluation, Configurational methods, Coincidence analysis, Implementation, Context

## Abstract

**Background:**

The Department of Veterans Affairs (VA) is the largest integrated healthcare system in the United States and serves a rapidly aging patient population. The VA’s Home-Based Primary Care (HBPC) program is a home care model for older, complex, high-risk Veterans that provides comprehensive, longitudinal primary care delivered by an interdisciplinary team of VA staff, with plans for expansion by 2027. HBPC program implementation varies considerably across local sites and contexts. Accordingly, these characteristics offer a unique opportunity to investigate real-world, cross-case heterogeneity and identify crucial factors that may lead to improved patient outcomes.

**Methods:**

Patient home time in the last 180 days of life will serve as the primary outcome measure, calculated at the patient-level as the total days not in an acute care (e.g., emergency department [ED], hospital), post-acute care (e.g., skilled nursing facility), or institutional long-term care setting (e.g., nursing home) in the 180 days prior to death. Secondary outcomes include patient satisfaction and other utilization outcomes: ED visits, hospitalizations, days in long term care, care transitions, hospice use prior to death, and site of death. Multiple datasets will be combined to obtain a comprehensive view of HBPC site characteristics. This study will use an exploratory sequential mixed methods design to describe the heterogeneity in HBPC program implementation and evaluate how contextual factors and program delivery patterns influence home time and other patient outcomes. Analyses will identify difference-making configurations of contextual, operational, cultural, and care delivery factors that distinguish high- versus low-home time sites. Analytic methods include quantitative descriptive analyses, regression analyses, in-depth qualitative case studies at 10 high and 10 low home time sites, qualitative cross-case analysis, and the application of Coincidence Analysis to identify successful HBPC program configurations.

**Discussion:**

Health care systems can take advantage of real-world heterogeneity in program implementation across sites that vary in context and setting to identify the key difference-makers and use these findings to inform future program expansion. By understanding which HBPC program features lead to improved outcomes for older adults in specific contexts, the VA can learn where site variation is a positive sign of adaptation versus a sign of inefficiency and an opportunity for improvement.

**Clinical Trial Number:**

Not applicable.

**Supplementary Information:**

The online version contains supplementary material available at 10.1186/s12877-025-06502-7.

## Background

The Department of Veterans Affairs (VA) is the largest integrated healthcare system in the United States, and it cares for a rapidly aging patient population. By 2040, 46% of Veterans are expected to be over 65 years old, 7% over 75, and 10% over 85 [1]. Older Veterans are more complex medically and socially compared with younger Veterans and experience higher rates of hospitalization, with many requiring long-term services and supports. VA spending on long-term care costs is projected to increase from $6.1 billion in 2017 to $14.3 billion by 2037, with the majority of expenses related to long-term institutional care [2].

The VA seeks to provide eligible older Veterans with home and community-based services, aligned with organization-wide goals of allowing Veterans to age in place, providing care consistent with Veterans’ preferences, and reducing costs [3]. The VA’s Home-Based Primary Care (HBPC) program is a home care model for older, complex, high-risk Veterans that provides comprehensive, longitudinal primary care delivered by an interdisciplinary team of VA staff [4]. The program has grown from serving 9,000 Veterans in 2003 to 55,000 in 2023. It is available at 139 out of 170 VA medical centers along with satellite teams in over 300 Community-Based Outpatient Clinics (CBOCs). Patient enrollment in HBPC is associated with improved patient outcomes, less hospital use, lower costs, delayed or prevented long-term institutionalization, and improved end of life care [5–8, 8, 8, 10, 11]. Given an increasing need for comprehensive geriatric services to care for an aging Veteran population, the VA plans to expand HBPC by an additional 75 sites by fiscal year 2026 [12].

HBPC program implementation varies considerably across local sites and contexts. Accordingly, it offers a unique opportunity to learn from real-world heterogeneity by applying rigorous methods to analyze existing cross-site variation and identify the crucial differences in patterns of factors that lead to improved patient outcomes [13, 14]. The objectives of this study are to: (1) describe the heterogeneity in HBPC program implementation; (2) evaluate how contextual factors and program delivery patterns influence patient outcomes, such as home time; and (3) identify difference-making configurations of contextual, operational, cultural, and care delivery factors that distinguish high- versus low-performing sites. This manuscript adheres to the SQUIRE guidelines and describes the methods used to conduct this HBPC program evaluation [15].

## Methods

### The Home-Based primary care program: a complex intervention in a complex system

HBPC targets older patients with chronic, serious, medical, social, or behavioral conditions that put them at high risk of hospitalization, nursing home placement, emergency care, and poor clinical outcomes [16]. The main goals of HBPC are to provide comprehensive, integrated care to maximize or maintain function; minimize institutionalization; maintain quality of life; and, in the case of terminal illness, provide and coordinate end-of-life care consistent with the Veteran’s wishes, including dying at home. There is wide variation across sites in terms of program structure and staffing arrangements, numbers of patients enrolled, and patient characteristics (e.g., demographics, prevalence of chronic conditions, hospitalization risk, frailty, caregiver supports, functional deficits). HBPC programs also have varied priorities that adapt to local needs. Some function as longitudinal home care programs while others primarily provide transitional care to reduce hospital readmission or palliative care for Veterans with limited life expectancy.

HBPC is a complex health intervention defined by the presence of multiple interdependent components embedded in multilevel complex systems [17]. As such, individual HBPC sites interact with and co-evolve with their local environments and the populations they serve. Complex, non-linear systems such as HBPC are difficult to study with traditional analytic methods because of multiple interactions among variables, feedback loops, path dependency, and contingencies [18–20]. Widely-practiced research approaches often seek to dissect a complex system into parts in order to understand and optimize its components, with an overall goal of improving system performance [21]. However, in a complex system, the interrelated nature of the components is a defining characteristic, and examining components independently often yields null results. The whole is greater than the sum of its parts, and studying complex systems needs a holistic approach that attends to interactions between components and change over time [22, 23]. Complex systems require methodological approaches with the capacity to analyze the interplay of components, including the ways that specific components work as difference-makers only when they occur together with other components or conditions, as well as the ability to identify when multiple pathways might lead to the same outcome (e.g., the pathway for a large urban teaching hospital may differ from the pathway for a more rural medical center) [24].

### Conceptual framework

A conceptual framework drawing upon the literature on patient complexity [25, 26], Wagner’s chronic care model [27], and Allen’s care trajectory management framework [28] will guide data collection and analysis. The components of the conceptual framework are provided in Table 1. Fixed contextual factors include issues such as urban versus rural location; region within the United States; complexity of the VA sites, including the clinical programs that are offered; and alternative programs for complex patients, such as geriatrics-focused primary care teams (Geri-PACT).

**Table 1:** Conceptual framework domains

Domain	Components
Fixed Contextual Features	Hospital versus community-based outpatient clinic Urban versus rural setting Facility complexity Region (State) Implemented new VA Electronic Health Record Other programs for high-risk patients Constraints/Policies
Modifiable Operational Factors	Physician versus nurse practitioner led team Primary care co-management Staffing model Program size Admission and discharge processes Use of predictive data tools for patient selection Communication practices
Modifiable Team Cultural Factors	Team priorities Psychological safety Sensemaking Boundary spanning Adaptive capacity Relationship-centered orientation
Modifiable Care Delivery Patterns	Patient selection Admissions per year Visit frequency Range of disciplines Telehealth use Length of Stay
Patient Characteristics	Medical and mental health complexity Functional limitations Social complexity

Modifiable operational factors include several key features. For example, staffing models vary across sites. The national VA geriatrics policy specifies that HBPC programs consist of an interdisciplinary team including a primary care provider, nurse, pharmacist, social worker, rehabilitation therapist, mental health provider, and dietitian, in addition to a program director, medical director, and administrative support. However, some sites do not have all roles staffed. HBPC teams can be structured with physician or nurse practitioner leadership. Some HBPC programs allow for patient co-management by clinic-based primary care providers [13]. Modifiable operational factors also include inter and intra-team communication practices, admission and discharge processes, patient selection practices including use of predictive data tools to select high-risk patient who may benefit most from HBPC [29–32].

Modifiable team cultural factors include team goals and priorities, psychological safety, sensemaking practices [33, 34], boundary spanning across VA services and with external systems [35, 36], relationship-centered orientation [37–39], and adaptive capacity to customize care over time and in the face of changing patient needs.

Modifiable care delivery components include methods of patient selection, visit frequency, length of stay within the program, range of HBPC disciplines that see patients, and integration of telehealth technologies [40].

### Patient cohort

We will identify Veterans who were enrolled in HBPC during the period of October 1 st, 2020 to September 30th, 2021. We expect to include approximately 60,000 patients in the cohort, cared for within a total of 440 HBPC programs, with the majority of patients in the range of 69–87 years of age, based on preliminary studies on the HBPC population in FY15 [41].

### Outcome measures

Given the age, medical complexity, and evolving goals of HBPC patients, choosing appropriate outcome measures for program evaluation presents a challenge. Many patients are near death, and mortality is not necessarily a negative outcome. Hospitalization is also not always a negative event if hospital care of an acute event is the most effective and patient-centered approach.

As maximizing patients’ time living at home independently with an acceptable quality of life is a universal goal in HBPC, measurement of patient home time will serve as the primary outcome measure [42, 43]. Home time will be calculated at the patient-level as the total number of days not in an acute care (e.g.,ED, hospital), post-acute care (e.g., skilled nursing facility), or institutional long term care setting (e.g., nursing home) in the last 180 days of life among HBPC decedents.

Secondary outcomes include patient satisfaction and utilization outcomes, including the number of ED visits without hospitalization, number of hospitalizations, and hospice use will be summarized separately as binary variables indicating any utilization within 1 month, 6 months, or 12 months of HBPC enrollment, and also any utilization between enrollment and the end of fiscal year 2023. Other outcomes include the number of care transitions (number of transitions between care settings), days in long term care and site of death. All measures —with the exception of patient satisfaction, which is measured at the site level—will be measured at the patient level and aggregated to the site level for comparison.

### Independent variables

In addition to data that describes site-specific contextual factors, modifiable operational factors, and modifiable care delivery patterns, we will collect data on patient factors including: demographics (gender, age, race, and ethnicity); social deprivation index of Veteran’s home location [44, 45]; comorbidities; functional status including deficiencies in activities of daily living (transfer, eating, walking, bathing/showering, dressing, using toilet); mobility deficits (assistance with mobility, bed disabled); sensory deficits; caregiver characteristics; marital status; living situation (alone versus with family versus group setting); risk scores such as the JEN Frailty Index (JFI) [46], Medicare Hierarchical Condition Category (HCC) V21 scores, Nosos score, and Probability of Long Term Institutionalization (PLI) score, and the Clinical Assessment Needs (CAN) score which predicts the probability of mortality and hospital admission [47]. We also plan to perform a latent class analysis at the patient level, examining combinations of comorbidites and functional deficits, to generate patient subgroups that can be used in subsequent analyses.

### Data sources

Multiple unique datasets will be combined to obtain a comprehensive view of HBPC site characteristics, HBPC patient characteristics, and clinician practice patterns. Supplemental Table A shows key variables and data sources. The HBPC Masterfile contains intake data from all Veterans admitted to HBPC including enrollment date, activities of daily living, sensory deficits, and presence of caregiver supports. The Geriatrics and Extended Care (GEC) Data & Analysis Center GECDAC Core Files (GCF) includes detailed data on patient clinical characteristics (demographics, clinical conditions), risk scores and GEC program service use. The GCF is constructed from a range of data sources including: VA inpatient and outpatient files; VA fee basis files (for care provided in the community but paid for by the VA); the VA Corporate Data Warehouse; VA enrollment files; VA vital status file; Managerial Cost Accounting; HBPC Masterfile; the Centers for Medicare & Medicaid Services (CMS) beneficiary summary files; CMS inpatient and outpatient claims; and the Planning System Support Group Distance File. The Residential History File (RHF) provides patient-level longitudinal data on acute, post-acute and long-term care including ED visits, hospital stays, skilled nursing facility care, nursing home and hospice care, as well as HBPC enrollment and Medicare Home Health Care use [48]. The RHF is based upon VA inpatient and outpatient records, fee basis files, vital status, managerial cost accounting data, HBPC Masterfile, Medicare and Medicaid claims, and Resident Assessment Instrument data from the Minimum Dataset required for all nursing home admissions. Corporate Data Warehouse (CDW) data includes stop codes to identify encounters specific to each HBPC discipline. Consumer Assessment of Healthcare Providers and Systems Home Health Care Survey (HHCAHPS) includes bi-annual surveys of a random sample of Veterans enrolled in HBPC.

### Analytic approach

The goals of this study are to describe the heterogeneity across HBPC program implementation and then identify the difference-making configurations of contextual, operational, and cultural factors that link directly to improved patient outcomes. This study will use an exploratory sequential mixed methods design [49, 50].

### Quantitative descriptive analyses

We will begin the study with descriptive quantitative analysis, examining HBPC sites in terms of contextual factors, operational factors, care delivery patterns, and patient characteristics. We will create displays of patient trajectories across care settings using heatmaps and Sankey plots, recoding daily site of care for each patient beginning at the date of enrollment in HBPC and ending on September 30, 2023.

### Regression modeling to identify factors associated with outcomes

Next, we will conduct a series of regression analyses to understand the relationship between modifiable operational factors and patient outcomes (e.g., home time), controlling for contextual and patient factors. Because we anticipate a slightly skewed left distribution for home time, we will use Tobit mixed effects models [51] to assess the association of home time with modifiable operational factors and care delivery patterns. Analyses will be conducted at the patient level with random effects for site to account for nesting of patients within sites. We will run separate models for each predictor of interest, patient characteristics, and site contextual features as covariates. Because home time varies by region [52], we will obtain home time benchmark data in the last six months of life for Medicare decedents in regionally-matched hospital referral areas to use as a regional adjustment variable. If the correlation between home time among Medicare decedents and HBPC users is low, we will add variables for additional institutional supply factors (e.g., hospital or nursing home density) to models to account for regional variation in home time. For the secondary outcomes, utilization measures are either binary (e.g., death, hospice use) or count (e.g., number of ED visits, acute hospitalizations, care transitions). These will be analyzed using logistic or negative binomial generalized estimating equations (GEEs), with clustering within sites. Patient satisfaction is a site-level variable continuous on a scale from 1 to 100, and thus analyses will be site- rather than patient-level, with average patient values at a site for covariates. Based on the satisfaction distribution shape, we will use either multivariable linear regressions with or without a transformation or Tobit models if there is a strong ceiling effect due to high satisfaction levels.

As an exploratory analysis, we will use a model-based cluster analysis to examine the association between combinations of site factors and patient outcomes. Specifically, we will use the unsupervised learning method model-based clustering which uses Gaussian mixture models to identify clusters of HBPC sites that are similar to each other. Model-based clustering will be run using the R package mclust [53]. To check for consistency of HBPC site clusters, we will compare the results with those from other unsupervised clustering techniques, such as k-means (centroid-based) agglomerative hierarchy clustering, and spectral clustering. Once clusters of sites have been identified, we will compare contextual factors, delivery patterns, and patient characteristics between clusters using descriptive statistics and unadjusted statistical tests (such as ANOVA and chi-squared) to identify which are most associated with the clusters and also whether there are differences in the outcomes between clusters. All analyses will be performed in RStudio using the newest version of R (version 4.5.0) [54].

### Identify high- and low- home time sites

We will examine the site level distribution of site level home time in the last 180 days of life and purposively sample 10 high home time sites from the top quartile and 10 low home time sites from the bottom quartile. We will stratify sampling by site level HCC quintile, patient level LCA subgroups based on comorbidities and functional status, patient rural/urban residency and geographic region. This sampling strategy will ensure different configurations of care practices, constructed to meet diverse patient needs, are observed and included in the sample.

### Qualitative methods: cases studies of high- and low-home time sites

Next, we will use qualitative methods to assess HBPC team member and Veteran perspectives on potential mechanisms of success in high- and low-performing sites by conducting in-depth case studies of HBPC sites at the ten high- and ten low-performing sites. Our qualitative approach will be grounded in a realist paradigm that assumes multiple pathways to the same outcome are possible and likely (equifinality of outcomes) and intrinsically shaped by context [55]; it is therefore necessary to consider context rather than to eliminate or control for it in study designs. The approach will also be realist [56] in occupying an epistemological position between positivism and constructivism: participants’ interpretations of how HBPC “works” will be brought together with data collected and observed by the research team, with both contributing to an imperfect but more complete understanding of program performance. We will collect data on HBPC operational and cultural factors using a program summary form, observation of team meetings, semi-structured interviews with program directors, staff (HBPC director and nurse), as well as Veterans and their caregivers. Interview guides will include open ended questions about HBPC goals and function, and deductive questions about contextual, operational, and cultural factors. Interviews will be audio-recorded and transcribed. We will conduct qualitative thematic analysis using a hybrid inductive/deductive approach to the coding of data and identification of findings [57]. An initial coding scheme for the analysis of the interview transcripts and meeting field notes will be developed with deductive codes (based on the domains in our conceptual model) and inductive codes iteratively developed as the research team reads, reflects on, and discusses the transcripts and field notes. This use of a “living codebook” approach will allow for iterative code development throughout the qualitative analysis. Each interview and set of notes will be coded by at least two members of the qualitative analysis team using Atlas.ti ^®^ software. Differences in coding between the two coders will be discussed as a team to facilitate building consensus on the definition of codes and their application. Potential themes will be noted, and new codes iteratively developed, as well as new questions or probes to add to interviews. Team members will also write a brief case summary highlighting distinctive aspects of the interview as a whole. Deductive coding will apply preidentified domains for categories with potential to be “optimized”: constraints, standardized admission/discharge processes, use of predictive data tools, communication practices, sensemaking, psychological safety, boundary spanning, relationship centered orientation, and adaptive capacity. For each category, we will develop 2–5 categories of possible responses (e.g., present/absent, full/partial/none, high/medium/low/absent, etc.) that represent the findings from the qualitative data for each site. We will search for patterns between and across high and low home time sites to identify what constitutes an optimized presence of a domain characteristic. Depending on what we learn, we may alter the domains we choose to categorize and the response categories within those domains.

### Mixed methods: cross-case analysis

We will conduct a cross-case analysis using manual sorting and pattern finding [58]. We will integrate data from the quantitative site summaries and the coded qualitative data in a case-by-attribute matrix for each HBPC site in our sample. We will sort this matrix on a range of attributes, including outcomes and structural characteristics to examine relationships between variables and outcomes.

### Mixed methods: configurational analysis to identify difference-makers that distinguish high- versus low-home time HBPC sites

Configurational Comparative Methods (CCMs) are mixed methods approaches well-suited to handling both complexity and context when evaluating HBPC. CCMs are case-based comparative methods that use a set-theoretic approach to examine the relationships between combinations of contextual and program features with outcomes. CCMs uses Boolean algebra to systematically examine sets of program and contextual factors to identify a crucial set of difference-makers that uniquely distinguish cases with the outcome (such as high-home time sites) from those without (such as low-home time sites) [59–63]. CCMs use a regularity framework of causality, which is a fundamentally different logic of inference than correlation-based methods such as linear regression [64]. Major advantages of this approach include its versatility with small-n studies; its capacity to model conjuncts, when several conditions must jointly appear for cases to have the outcome of interest; and its ability to identify disjuncts, when multiple pathways lead to the same outcome [65]. Additionally, positive models (solutions where the outcome is present) are developed separately from negative models (solutions where the outcome is absent) [61, 63, 65–67]. For studying complex interventions embedded in multilevel systems like HBPC, CCMs can consider factors at multiple organizational levels in the same analysis. CCMs have increasingly been applied in health services research and implementation science [24, 67–72].

In this evaluation of HBPC, we will apply a specific configurational method called Coincidence Analysis (CNA), a relatively new member within the larger family of CCMs. Coincidence analysis (CNA) is a configurational comparative, case-based method that uses Boolean algebra, logic, and set theory to identify conditions that uniquely distinguish a group of cases with an outcome from another group of cases without that outcome [65, 73]. CNA offers several advantages: it includes a data-driven factor selection process, models are by definition redundancy-free, and it can identify causal chains in a dataset if they exist [65, 74]. At present, there are over 100 CNA-related publications in the peer-reviewed literature, mostly published within the past four years. All CNA analyses will be conducted using the latest version of R, RStudio, and the R package “cna” (version 3.6.2) [75]. As is standard in configurational analysis, we will conduct CNA analyses separately for the presence and absence of the outcome [65].

We will use multi-value CNA to integrate our quantitative and quantified qualitative data with the analytic target of identifying contextual and modifiable operational and cultural difference-makers that uniquely distinguish high home time from low home time sites (as well as vice-versa). The CNA analyses will begin by meeting with operations partners to review qualitative findings from the case summaries as well as quantitative results from the descriptive and modeling analyses to identify an initial set of factors to include as part of the exploratory data analysis phase. We will then assemble this set of initial factors to form the analytic dataset for multi-value can [76].

Next, the “minimally sufficient conditions” (msc) routine within the R package “cna” will be used as part of an exploratory data analysis to inform the selection of a smaller set of candidate factors to carry forward into the modeling phase [59, 77, 78]. The msc routine is an established method within CNA to comprehensively scan an entire dataset and identify configurations of specific conditions with especially strong connections to an outcome of interest. Specifically, the msc routine will be run multiple times at different prespecified consistency thresholds (95%, 90%, 85%, 80%, and 75%), exhaustively considering all possible one-, two-, and three-condition configurations instantiated within the analytic dataset to see if they met the consistency threshold [77, 78]. Next, configurations that meet specific thresholds will be retained and organized into a “condition table,” where rows represent individual configurations and columns represent values for the outcome, conditions, consistency, coverage, and complexity. Consistency is a measure of reliability, calculated as the number of cases that are both identified by the configuration and have the outcome present divided by the total number of cases that are identified by the configuration. Coverage is a measure of explanatory breadth, calculated as that are both identified by the configuration and have the outcome present divided by the total number of cases with the configuration present. Complexity represents the number of conditions within a configuration [62, 65].

We will then review the condition table to identify configurations that meet al.l of the following four criteria: (1) top coverage score within configurations of the same complexity level (i.e., same number of conditions); (2) clear separation in coverage score between the top-scoring configuration and its next nearest neighbor; (3) relevance to the research question; and (4) aligned with logic, theory, and prior knowledge. Using this approach, we will inductively analyze the entire analytic dataset during this stage of the exploratory data analysis and identify a smaller subset of factors to carry forward into model iteration, with that factor selection process informed both by the mathematical output generated by the msc routine as well as our own substantive knowledge, case familiarity, and theoretical understanding [77].

During the modeling phase of the CNA analysis that follows, our goal will be to develop models which explain ≥ 80% of cases with the outcome (i.e., coverage) and yield the outcome ≥ 80% of the time (i.e., consistency). Preliminary CNA models will be shared with operations partners for review and feedback, with these comments leading to further model refinement and development as needed.

### Sample size

For the regression analyses of the primary outcome of home time, even with a very low estimate of 11,000 patients at 440 HBPC sites, the effective sample sizes (ESSs) for intraclass correlation coefficients (ICCs) 0.01–0.05 are 5000–8871. With the smallest ESS of 5000 (ICC = 0.05) and comparing 6 groups in a one-way ANOVA, we have 90% power to detect a Cohen’s f effect size of 0.07, when alpha = 0.005 (applying a Bonferroni correction for 10 tests). A Cohen’s f of 0.1 is considered to be small, and f of 0.25 is considered to be medium. The staffing model predictor has the most comparisons groups at 6, and other predictors with fewer comparison groups will have even greater power. Power analyses were run using PASS 2022 [79]. For the qualitative analyses, a sample of twenty sites should provide adequate information for face validity as well as thematic saturation. For the CNA analyses, twenty cases with ten high- and ten low-home time sites should provide sufficient cross-case variation to identify difference-making configurations.

## Discussion

As part of the VA’s commitment to embodying the principles of an age-friendly health system, the evidence-based HBPC program has plans to expand by an additional 75 sites by 2026 [12, 80]. The success of the HBPC expansion will depend on understanding what HBPC program features lead to improved outcomes in specific contexts. This includes learning where site variation is a positive sign of adaptation versus where it is a sign of inefficiency and an opportunity for improvement. This study aims to provide critical insights needed for HBPC expansion efforts as well as lay the groundwork for future adaptive quality improvement interventions.

Two strengths of the planned approach are noteworthy. First, the use of patient home time as the key outcome measure will provide a metric to evaluate performance on the basis of a construct that captures what genuinely matters to patients: living independently at home as long as possible. Home time has been associated with positive patient-reported outcomes [81] and is increasingly being adopted as an outcome measure [42, 52, 82, 83]. Second, the use of configurational methods allows for a mixed methods blending of quantitative and qualitative data that has the potential to uncover a variety of successful HBPC configurations. Because CNA may produce a model with equifinality, a variety of configurations of context, operational factors, and delivery patterns may be identified as be associated with good outcomes (i.e., high risk-adjusted home time). For example, sites in rural locations (fixed contextual factor) may have good outcomes if visit frequency is high (modifiable care delivery pattern) but may have poor outcomes if the staffing model is incomplete (modifiable operational factor).

Implementation of HBPC is growing outside of VA [84–86]. Although programs in the community face different challenges in program implementation, they similarly need to tailor programs to specific settings. Therefore, these research findings are also likely to be relevant to non-VA HBPC practices.

Although the study has been designed to address key gaps in knowledge, several limitations merit description. First, the study will be conducted within the organizational setting of the VA, which has a robust electronic health record system, a culture of quality improvement, and a commitment to serving as a learning health system [87]. Accordingly, care should be taken when generalizing results from this study to other health care systems. Second, our qualitative and configurational analyses will be conducted at 20 sites selected on the basis of the home time outcome, the modeling results, and stakeholder input. These sites may not represent the full spectrum of HBPC implementation experience. Finally, the quantitative data will use data from 2021 to 2022 to allow for complete data from all sources, so findings may not reflect more recent changes in HBPC program implementation.

In conclusion, this study will provide an exemplar of how a national health care system can apply rigorous methods to learn from existing cross-site heterogeneity in program implementation to identify key difference-makers. By understanding what HBPC program features lead to improved outcomes in specific contexts, the VA can learn where site variation is a positive sign of adaptation versus where it is a sign of inefficiency and an opportunity for improvement. This study aims to provide critical insights needed for HBPC expansion efforts as well as lay the groundwork for future adaptive quality improvement interventions.

## Supplementary Information

Supplementary Material 1.

## Data Availability

A de-identified dataset for this study will be available upon request at the completion of data collection and analysis.

## Abbreviations

ANOVA: Analysis of Variance
CAN: Clinical Assessment Needs
CCMs: Configurational Comparative Methods
CDW: Corporate Data Warehouse
CLC: Community Living Center
CMS: Centers for Medicare & Medicaid Services
CNA: Coincidence Analysis
ED: Emergency Department
ESS: Effective Sample Sizes
GCF: GECDAC Core Files
GEC: Geriatrics and Extended Care
GECDAC: Geriatrics and Extended Care Data & Analysis Center
GRECC: Geriatric Research, Education, and Clinical Center
HBPC: Home-Based Primary Care
HBPC MF: Home-Based Primary Care Masterfile
HCC: Hierarchical Condition Category
HHA: Home Health Aide
HHCAHPS: Consumer Assessment of Healthcare Providers and Systems Home Health Care Survey
ICC: Intraclass Correlation Coefficients
JFI: JEN Frailty Index
LTSS: Long Term Services & Supports
PACT: Patient-Aligned Care Team
PCP: Primary Care Provider
PLI: Probability of Long Term Institutionalization
RHF: Residential History File
SQUIRE: Standards for Quality Improvement Reporting Excellence
VA: The Department of Veterans Affairs
