# Supplementary material for: Defining successful program configurations in VA home-based primary care: a study protocol to identify key difference-makers through investigating cross-case heterogeneity in program implementation
Source: BMC Geriatr. 2025 Nov 25;25:1049. doi: 10.1186/s12877-025-06502-7 (PMC12751214; doi:10.1186/s12877-025-06502-7)
Supplement: Supplementary file 1 — Supplementary Material 1. [file 12877_2025_6502_MOESM1_ESM.docx]

**Supplemental Table A. Variables and Data Sources**

| **Domain** | **Source***** | **Definition** | **Level** |
| --- | --- | --- | --- |
| **Outcomes** | | | |
| Home Time (Primary Outcome) | RHF | Days not in institutional care in last 180 days of life* | Patient** |
| Emergency Department visits | RHF | VA or Medicare | Patient** |
| Hospitalizations | RHF | VA or Medicare | Patient** |
| Care Transitions | RHF | Number of transitions between care settings | Patient** |
| Days in Long Term Care | RHF | Days in Skilled Nursing Facility or Nursing Home | Patient** |
| Hospice use prior to death | RHF | Any VA or Medicare hospice use among HBPC decedents | Patient** |
| Site of Death | RHF | Site of care immediately prior to death among HBPC decedents [9] | Patient** |
| Patient Satisfaction | GEC | HHCAHPS | Site |
| **Fixed Contextual Factors** | | | |
| VAMC vs CBOC | GEC | Hospital based vs. Outpatient clinic based site. | Site |
| Urban vs. Rural | GEC | HBPC Site Location, based on RUCA coding | Site |
| Region | CDW | Region within United States (Northeast, West, South, Midwest) | Site |
| Facility Complexity | VSSC | VA facilities classified into 5 categories of complexity: 1a, 1b, 1c, 2, 3 | Site |
| Implementing new EHR | CDW | Implementing Cerner Millenium EHR | Site |
| Other GEC Programs | GCF | Presence of Geri-PACT, GRECC, CLC | Site |
| **Modifiable Operational Factors** | | | |
| Physician vs. NP led team | GEC | Profession of primary HBPC clinician | Site |
| Co-management with clinic based primary care | CDW | Care from clinic-based PCP and HBPC providers while enrolled in HBPC | Site |
| Staffing Model | CDW | Full interdisciplinary team, Core Team, Core Team +, Home Health Agency (HHA), HHA+, House Call Practice [15] | Site |
| **Modifiable Care Delivery Patterns** | | | |
| New patients/year | HBPC MF | Annual admissions | Site |
| Visit Frequency | CDW | Frequency of HBPC Visits/Days Enrolled | Patient** |
| Telehealth Use | CDW | Telephone visits, video visits, asynchronous telehealth encounters | Patient** |
| Range of Disciplines | CDW | Provider, Nurse, Social Work, Pharmacy, Dietetics, Rehab | Patient** |
| HBPC Length of Stay | RHF | Days from HBPC admission to discharge | Patient** |
| **Patient Factors** | | | |
| Demographics | GCF | Age, Sex, Race, Ethnicity | Patient** |
| Latent Class Analysis derived patient subgroups | GCF, CDW, Medicare | Subgroups developed based on comorbidities and functional status. | Patient** |
| Risk Scores (HCC, Nosos, JEN Frailty Index, HNHR2, PLI, CAN) | GCF | Scores predict cost (HCC, Nosos), LTSS use (High-Needs High-Risk 2, JEN frailty index, PLI), hospitalization/death (CAN). | Patient** |
| Functional Status | HBPC MF | Activities of Daily Living, Sensory deficits, Communication Deficits | Patient** |
| Social Support | HBPC MF | Caregiver status, marital status, living situation | Patient** |
| Located in deprived area | GCF | Veterans home location coded using social deprivation index [88] | Patient** |

*Institutional Care defined as Emergency Department, Hospital, Skilled Nursing Facility, or Nursing Home

** Patient level variables summarized at site level in site level models and in Aim 3 configurational analysis.

***HBPC MF refers to the Home-Based Primary Care Masterfile, GCF to the Geriatrics and Extended Care Data & Analysis Center (GECDAC) Core Files, CDW to the VA Corporate Data Warehouse, RHF to the Residential History File (RHF), HHCAHPS to the Consumer Assessment of Healthcare Providers and Systems Home Health Care Survey, VA to the United States Department of Veterans Affairs, HCC to Medicare Hierarchical Condition Category V21 scores, PLI to Probability of Long Term Institutionalization, CAN to Clinical Assessment Needs score, LTSS to long term services and supports, Geri-PACT to geriatric patient aligned care team, GRECC to Geriatric Research, Education and Clinical Center, and CLC to community living center.
